# Investigating the Architecture and Characteristics of Asian Hornet Nests: A Biomimetics Examination of Structure and Materials

**DOI:** 10.3390/ma16217027

**Published:** 2023-11-03

**Authors:** Naim Sedira, Jorge Pinto, Mário Ginja, Ana P. Gomes, Miguel C. S. Nepomuceno, Sandra Pereira

**Affiliations:** 1 University of Trás-os-Montes e Alto Douro (UTAD), 5000-801 Vila Real, Portugal; tiago@utad.pt (J.P.); mginja@utad.pt (M.G.); spereira@utad.pt (S.P.); 2 C-MADE–Centre of Materials and Building Technologies, UBI, 6201-001 Covilhã, Portugal; 3 Centre for Animal Sciences and Veterinary Studies (CECAV), UTAD, 5000-801 Vila Real, Portugal; 4 University of Beira Interior (UBI), 6201-001 Covilhã, Portugal; anapaula@ubi.pt (A.P.G.); mcsn@ubi.pt (M.C.S.N.); 5 FibEnTech–Fiber Materials and Environmental Technologies, Optical Centre, UBI, 6201-001 Covilhã, Portugal; 6 Lab2PT, Landscape, Heritage and Territory Laboratory, 4800-058 Guimarães, Portugal

**Keywords:** biomimetics, construction, architecture, vespa velutina nigrithorax, eco-material, CT scan, SEM-EDS

## Abstract

This study investigates the internal architecture of Asian hornet nests (AHNs) using advanced imaging techniques, such as CT scanning and X-ray radiography, to understand their construction and function. The primary objective and significance of this study centre on drawing inspiration from the creative way Asian hornets construct their nests, with a particular focus on the architecture, design, functionality, and building materials of these nests. The architectural principles governing the construction of these nests, such as the arrangement of hexagonal cells, pedicels for load bearing, and adhesive materials, serve as a source of inspiration for innovative and sustainable design practices. The pedicels in Asian hornet nests play a crucial role in transferring load and ensuring stability. Additionally, AHNs’ adhesion to tree branches is essential for preventing collapse, and the pedicels provide necessary structural support. The knowledge gained from studying AHNs’ internal architecture could be applied directly to the architecture and civil engineering fields to improve structure stability and durability. The microstructure analysis of the paper-like material that hornets produce to build their nests indicates a complex and heterogeneous structure, composed of various plant fragments and fibres. This unique composition creates intricate grooves and pores, which are essential for regulating temperature and humidity levels within the outer envelope of the nest. The study of Asian hornet nests’ internal structure demonstrated that nature’s engineering principles inspire the design of durable and resilient structures in the construction industry. Civil engineers can incorporate similar principles into their designs to enhance the structural integrity and performance of buildings, bridges, and other infrastructure.

## 1. Introduction

Nature offers instances of robust materials and structures that possess optimised morphologies and topologies, thereby enabling exceptional mechanical and structural properties. These instances present sustainable alternatives for the construction sector [1]. Animal architecture is a fascinating topic in natural history, which serves animals’ survival by providing protection against predators and external elements. In particular, social insects’ nests play a crucial role in colony life, providing valuable information on colony size, growth rate, division of labour, food storage, defence, and economic considerations. Additionally, it is related to the concepts of extended phenotype and homeostasis, among other parameters that warrant further exploration [2,3]. Unlike other major social insect groups, social wasps typically build their nests above the ground, exposing them to the vagaries of the environment. These nests are engineered to sustain their weight by hanging from a substrate. Wasps manufacture a paper-like material by masticating pulp scraped from dead wood or plant hairs collected from living plants, combined with bark, dead leaves, or other plant tissues from the forest floor [4].

Vespa velutina Lepeletier, 1836 (Hymenoptera: Vespidae) is an Asian hornet native to the mountains of Southeast Asia, from Kashmir to Malaysia, spreading eastwards to Taiwan and south-eastwards to Sulawesi, the Sunda Islands, and Timor. There are 14 subspecies of this taxon [5,6], which differ from each other morphologically, primarily in their body colours. Among these, we have the Vespa velutina nigrithorax du Buysson, 1905 (South China) subspecies [7]. Vespa velutina nigrithorax has lately spread to Europe as an invasive species, and it is known for its large size, distinctive appearance, and aggressive behaviour. Like other Asian hornet subspecies, it is known for building large nests [8,9,10,11,12]. The literature on invasive social wasps has experienced significant growth in recent decades [13]. The invasion of the Asian hornet in most Western European countries highlights the need for targeted control measures to limit its economic, ecological, and social impact, underscoring the failure of current trapping strategies and the need to optimise nest detection techniques and investigate new control strategies [14]. The predation of European honeybees (Apis mellifera Linnaeus) by the Vespa velutina nigrithorax du Buysson invader is of significant concern due to its ecological impacts, such as the disturbance of pollination, as well as economic impacts, including monetary losses for beekeepers [15,16].

Asian hornets are social insects that live in a hierarchical community structure consisting of three classes: queens, workers, and males. The males have single copies of their species’ chromosomes (haploids), while the females have double copies (diploids). Towards the end of summer, multiple queens are produced from each nest and then mate. After mating with one or more males, the queen stores the sperm in an abdominal container. When the time is right, the queen lays fertilised and unfertilised eggs in large numbers, with a single queen capable of laying thousands of eggs and creating thousands of offspring. The original mated queen emerges during the initial warm days of spring and constructs the primary nest for the starting colony using materials such as wood pulp and leaves. The main Asian hornet nest (AHN) is typically compact and circular. It is located in a secure area where food is easily accessible, particularly honeybees, as they are a vital part of the larvae’s diet. After 4–5 weeks, the first female workers emerge and assist the queen. At this point, the wasps often leave the primary nest and construct a larger secondary nest on top of trees. The colony experiences growth during the summer, with the population increasing to thousands. Newly mated queens depart from the nest to establish a new colony elsewhere. The abandoned nest is likely to disintegrate during winter; however, a recent study found evidence of surviving individuals from all castes, as well as eggs and broods, inside the nests. Secondary nests should be removed during the winter to eliminate any residual colonies that may regrowth in the spring [10,17,18,19]. The annual life cycle of the Vespa velutina involves the emergence of workers following the construction of an embryo nest by a single founder queen under favourable environmental conditions, characterised by high temperatures and abundant food resources [20,21]. Social hornet nests are typically made of a paper-like material created from chewed wood and plant fibres and are usually found in trees. However, they can also be located in other structures, such as buildings or sheds. The size and architecture of the nest will vary depending on the colony’s size, with larger colonies having bigger and more complex nests [22]. Adopting environmentally friendly practices such as maintaining, reusing, and utilising natural or eco-building materials, as well as reducing water and energy consumption, can promote sustainable construction. Furthermore, the exploration of innovative building techniques, such as prefabrication, while taking logistical considerations into account, can also prove to be beneficial. In the construction industry, efforts are being made to develop fully intelligent and sustainable buildings that prioritise factors such as water and energy consumption, air quality, and acoustics. In order to achieve these goals, it may be beneficial to take a closer look at the animal kingdom. When considering the short period of time (a few months) and the size of the constructor (the hornet), the construction process of the Asian hornet nest is a remarkable achievement. The use of eco-friendly materials in this process provides further inspiration for the construction and material industries. A further study has been conducted in this area by the authors of the study [23,24]. The results of their study have shown that nest construction is an efficient process and provides many lessons for modern construction. Furthermore, the use of sustainable materials in the process is an example of how environmental considerations can be considered in construction to reduce the impact of construction on the environment and create a more sustainable future. The study conducted by Artem Holstov et al. [25] examines the feasibility of employing hydromorphic materials in adaptive building systems. The authors posit that the integration of such materials presents the possibility of creating architectural designs that are innately sensitive to the variable rhythms of both internal and external environments, thereby addressing a wide array of sustainability considerations. Through their research, the authors propose that a hydromorphic-based approach to design could not only improve a building’s energy efficiency but also enhance occupant comfort and health. Further, the Beijing National Stadium architectural design takes inspiration from a bird’s nest. This iconic structure is a symbol of modern Chinese architecture, blending traditional and contemporary elements. This is also seen in many other architectural projects that aim for sustainability and versatility by drawing inspiration from nature. These projects are designed to be energy efficient and use local materials as much as possible, creating a balance between the modern and the traditional. The Beijing National Stadium is an iconic example of this, showcasing the potential of nature-inspired design [26].

This study utilises a range of non-destructive techniques, such as CT scans and X-rays, to explore the intricate details of the architecture, shape, and size characteristics of an AHN. These methods enable researchers to examine both the internal and external structures of the AHN without causing any damage or alteration, thus ensuring the preservation of the nest and the retention of its intricate details for current and future studies and examinations. Additionally, the microstructure of nest-building materials was analysed using SEM-EDS. These findings have the potential to significantly contribute to future biomimetics studies, offering valuable information about the construction techniques used by Asian hornets. The implications of this research extend beyond the world of entomology, with applications in engineering, architectural design, and the development of innovative and sustainable materials for the building sector.

## 2. Materials and Methods

### 2.1. The Case Study of the Asian Hornet Nest

The Asian hornet nest in this study case is an oval-shaped structure with several centimetres in diameter. It is made of a paper-like material produced by the hornets using wood pulp and saliva [27]. The nest can be identified by its distinctive yellowish-brown colour and papery texture. AHNs are commonly found in trees and shrubs but can also be found on buildings or other man-made structures. AHNs tend to be more prevalent in warmer and humid climates. The physical characteristics of the studied AHNs are delineated in the following subsections.

#### 2.1.1. Nest Collection

**Figure 1:**
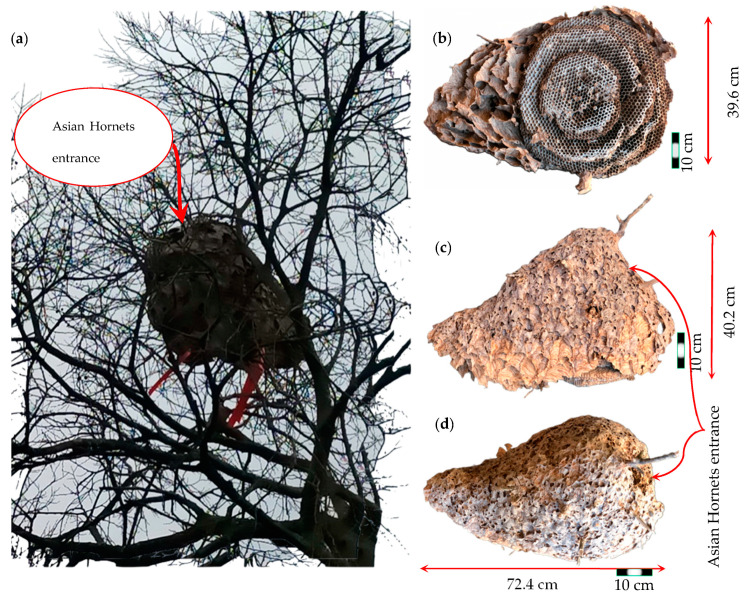
Large Asian hornet nest: (**a**) on top of tree; (**b**) inferior aspect; (**c**) side aspect; (**d**) superior aspect.

The current study focuses on an Asian hornet nest (AHN) that was situated atop a tree branch, as shown in Figure 1a, specifically a poplar tree (Populus) located in the city of Amarante in the Tâmega e Sousa subregion in northern Portugal. The AHN was removed from the aforementioned location via the severance of the main tree branch. Subsequently, Figure 1b–d portrays the multiperspective view of the AHN that has been preserved within a laboratory since December 2021, wherein controlled temperature and humidity have been maintained.

#### 2.1.2. Characterisation of the Aspects of the Asian Hornet Nest

The present study aimed to analyse and describe various aspects of an AHN. The Asian hornet nest in this case study has an oval-shaped structure. The disc-shaped comb is enclosed in an envelope, with a single entrance at the top of the outer envelope (the upper combs), as shown in Figure 1. Our research involved taking measurements of the nest’s weight, height, and diameter, as well as the number of combs present. The total weight of the nest considered both the weight of the structure (i.e., the outer envelope and combs) and the tree branch, while the total height referred to the height of the entire nest, including its outer envelope.

The measurements revealed that the nest had an overall length (*L_N_*) of 72.4 cm, a height (*H_N_*) of 40.2 cm, and a width (*W_N_*) of 39.6 cm. The weight of the nest was found to be 2.40 kg. The volume of the nest (*V_N_*) was calculated using the formula of a prolate spheroid (1):

(1) VN=43πLN22·WN2

*V_N_* = 61,261.7 cm^3^.

Given its considerable size, the nest was classified as a secondary AHN. Moreover, notable features, such as distinctive patterns or colouration on the nest’s exterior, were also observed. Specifically, the outer envelope of the nest exhibited a colouration that closely resembled the branch colour of the tree on which it was constructed. These findings could be significant in understanding the physical characteristics of AHNs and pave the way for future research.

### 2.2. Nest Architecture: CT and X-ray Scanner Analysis

Advanced imaging techniques such as CT and X-ray scans provide a thorough analysis of Asian hornet nests, revealing their internal structure in great detail. These non-destructive methods offer a unique opportunity to visualise the internal architecture of the AHN in unprecedented detail. One of the key benefits of using CT scans and X-rays to study hornet nests is that these methods provide detailed information about the nest structure, component arrangement, and functionality of each part in this complex structure.

#### 2.2.1. Computed Tomography (CT) Analysis

Computed axial tomography (CAT), also known as a computed tomography (CT) scan, is a radiological imaging technique that uses X-rays to analyse the internal structure of the Asian hornet nest (Vespa velutina nigrithorax) in a non-destructive manner [28].

Computed tomography (CT) assessment was performed using a Revolution™ ACT–16 slices scanner (General Electric Medical Systems, Hatfield, UK), using a helicoidal acquisition with a slice thickness of 1.25 mm.

**Figure 2:**
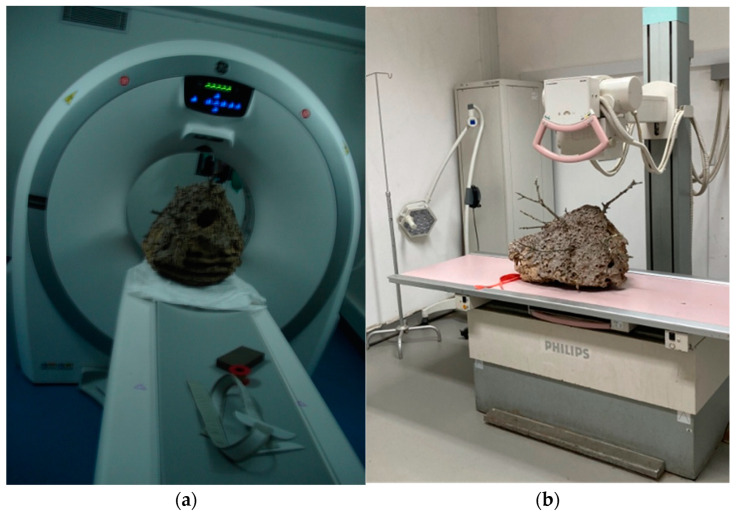
Equipment used for the examination of the Asian Hornet nest: (**a**) computed tomography (CT) assessment; (**b**) computed digital X-ray device.

CT scans create highly detailed, cross-sectional images of the nest, which can be reconstructed into a 3D model of its internal structure [29]. During the scan, the nest is placed on a rotating platform and imaged from various angles to generate a series of 2D images, as shown in Figure 2a. These images are then reconstructed into a 3D model using the Digital Imaging and Communications in Medicine “RadiAnt DICOM Viewer” software version 2022.1.1 [30]. The CT images provide information about the nest’s construction, including the size and location of combs and cells, the shape and size of the comb, and the different materials that make up the nest’s composition. The CT scan can also distinguish between the different parts of the nest, such as the outer envelope, the combs, the pedicels, and the tree branches.

#### 2.2.2. Computed Digital X-ray Analysis

The radiographic study was performed using an X-ray generator Philips Optimus 80 (Philips Medical Systems, Hamburg, Germany) and a computed digital X-ray device (Fujifilm, FCR Prima, Tokyo, Japan). Two perpendicular views were performed using an X-ray beam of 60 kVp and 5 mAs, with a focal distance of about 1 m and a 12:1 ratio moving grid. The Veterinary Hospital of UTAD conducted this analysis with meticulous attention to detail.

X-ray analysis is a non-invasive methodology that enables non-destructive investigation of the internal structure of an AHN without causing any disruption to its composition. During the X-ray analysis, the nest is strategically positioned on an X-ray plate and scanned from a specific angle, producing a two-dimensional image of the nest’s internal structure in two distinct positions, as depicted in Figure 2.

### 2.3. Microstructure Analysis of the AHN’s Paper-like Material

The Scanning Electron Microscopy (SEM) with Energy Dispersive Spectroscopy (EDS) analysis of the AHN’s paper-like material was conducted using small fragments cut from the outer envelope of the AHN, which were then observed using a scanning electron microscope (SEM-EDS) Hitachi S-4800 coupled with an EDX detector. The analysis was performed in the Optical Centre of the University of Beira Interior (UBI), Covilhã, Portugal.

SEM-EDS is a powerful tool for studying the microstructure and chemical composition of the AHN’s paper-like materials. SEM-EDS can reveal the morphological structure of the material and the chemical composition characteristics of the nest-building materials.

## 3. Results and Discussion

To obtain a thorough understanding of the internal structure of the hornet nest and to produce higher-quality 3D images, the data collected from the CT scan were processed using the “RadiAnt DICOM Viewer” software. The 3D volume rendering (3D VR) technique was employed to create detailed images, and the “Angio” parameter in the 3D presets tool was used to enhance the images further.

The 3D VR technique and the “Angio” parameter enabled a more comprehensive understanding of the nest’s internal structure, which is challenging to discern with conventional imaging methods. The “Angio” parameter, short for angiography, enabled the visualisation of the nest’s tissue structure. These tools proved highly effective for studying the intricate details of the hornet nest’s structure and function.

**Figure 3:**
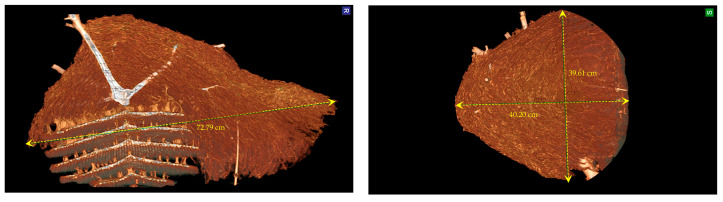
Asian hornet nest in different aspects and dimensions using CT scan, the patient orientation in space: (S)—superior and (R)—right.

The Vespa velutina nigrithorax hornet seems to have built its nest differently from other types of wasps in the region, enclosing the combs in an outer envelope to shield them from external elements (Figure 3). Each level of the comb is made up of hexagonal cells, which are used to hold the eggs, larvae, and pupae. Below, a detailed description is provided for the main constituent components of the AHN.

### 3.1. The Outer Envelope of the Asian Hornet Nest

The X-rays exhibit varying degrees of absorption by different types of tissue due to their differential electron densities, with denser tissues such as bone, metal, or tree branches absorbing a more significant amount of X-rays [31,32,33]. Low shading in a CT scan of an Asian hornet nest could refer to areas in the image where the density of the outer envelope of the nest appears to be low. Several factors, such as the presence of air pockets, empty spaces within the nest, and the lack of high-density structures in the nest, can cause this. Therefore, the outer envelope of the AHN is primarily composed of low-density materials such as paper or wood pulp. The resulting CT scan exhibits low shading due to the relatively lower attenuation of X-rays in these materials (which form the outer shell of the nest).

**Figure 4:**
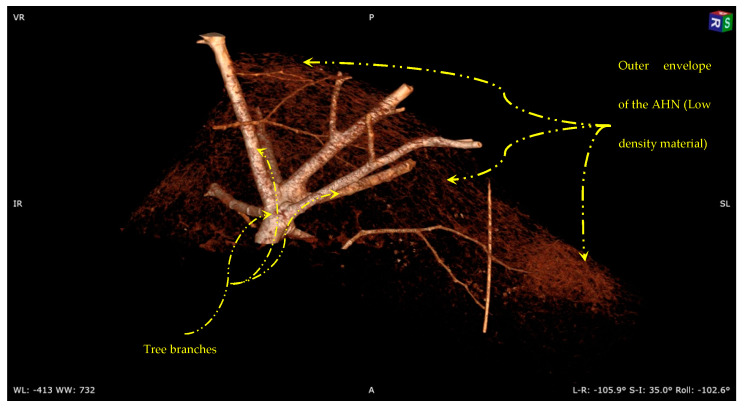
CT scan image shows the outer envelope of the Asian hornet nest (tree branches and paper-like material with low density), the patient orientation in space: (S)—superior and (R)—right.

**Figure 5:**
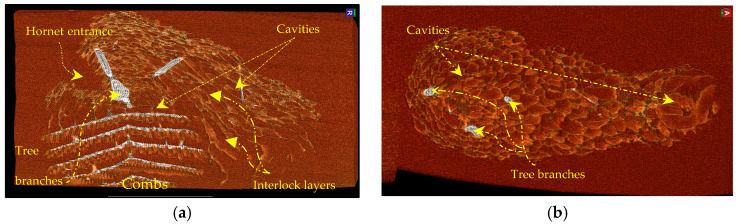
Outer envelope structure: (**a**)—longitudinal plane; (**b**)—coronal plane with increasing shading parameter, the patient orientation in space: (A)—anterior and (R)—right.

Figure 4 and Figure 5 illustrate the presence of overlapping and interlocking layers, forming a textured pattern that results in numerous interconnected cavities. The overlapping layers create protective cavities between the internal part of the nest and the external environment that minimise temperature variations. These cavities within the insulation layers vary in size and shape. In addition to this insulating role, the outer envelope (shield) has exceptional effectiveness in being exposed to sunlight for an extended period while protecting against UV radiation and rainwater. The denser nature of the upper-side skin, as illustrated in Figure 4, accounts for this phenomenon. The current study aimed to investigate the outer envelope of the AHN by increasing the shading parameter in the RadiAnt DICOM Viewer, as illustrated in Figure 5. In the outer envelope of the Asian hornet nest, a non-uniform distribution of cavities was observed. As one approaches the combs, the cavities in the outer envelope of the AHN become smaller in size but more numerous in quantity, resulting in a higher density of cavities (more densely packed). The shift of sizes and densities in the cavities significantly impacts the outer envelope protective function. The outer envelope protects the most pivotal component of the hornet nest (the combs) against UV radiation, sunlight, and rainwater infiltration [34]. The cavities present in the outer envelope of the hornet nests play a crucial role in enhancing insulation. These cavities create pockets of stagnant air that effectively impede heat transfer [35]. Consequently, the temperature within the nest can remain stable despite fluctuations in external temperature [36]. This insulation mechanism maintains optimal temperature conditions within the nest [37]. Based on the preceding information, it can be inferred that Asian hornet combs are encased in an exterior covering that performs diverse functions in sustaining the entire colony. This protective layer safeguards the nest against environmental threats, including UV radiation, rainwater, variations in temperature and humidity, and predators. Moreover, it helps to maintain optimal conditions for the growth and development of hornet larvae by regulating temperature and humidity levels within the nest [4]. In addition to providing a protective shield, the envelope functions as a structural support system for the hornet nest, furnishing stability and preventing potential collapse [38]. This is especially significant as the size of the nest can vary significantly. Therefore, the envelope must exhibit robustness and durability to sustain the nest’s structural integrity.

### 3.2. The Combs in the Hornet Nest

The combs are arranged in a symmetrical pattern. The shape of the combs is similar to that of mushrooms with an umbonate pileus, which have a rounded protrusion in the centre of their caps. The combs are a key structural component of a hornet nest, consisting of five series of parallel combs stacked vertically and held together by columns. The adhesion of the hornet nest to the tree branches is fundamental to preventing the collapse of the nest onto the ground.

**Figure 6:**
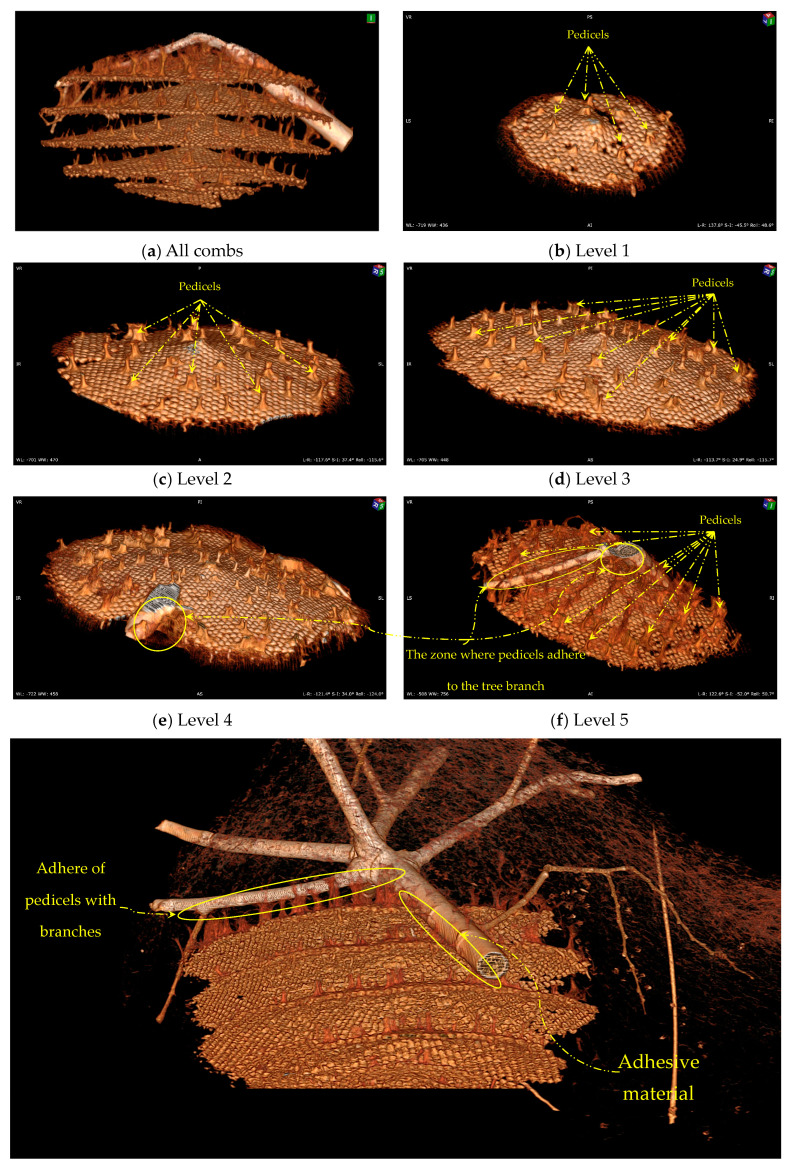
Combs of the Asian hornet nest: (**a**) overall combs; (**b**) 1st level comb; (**c**) 2nd level comb; (**d**) 3rd level comb; (**e**) 4th level comb; (**f**) 5th level comb, the patient orientation in space: (A)—anterior, (P)—posterior, (L)—left, (R)—right, (S)—superior, and (I)—inferior.

All the combs in the hornet nest are almost disc-shaped (Figure 6). This shape could have important implications for the nest’s structural stability and organisation. Circular combs could be able to distribute the weight of the nest more evenly, reducing stress on individual cells and the overall structure. This arrangement could be particularly important in larger nests, where the comb’s weight can significantly strain the nest-supporting pedicels and tree branches. Disc-shaped combs could also simplify the organisation of the nest, as each comb would be arranged in concentric circles around the nest’s central axis, making it easier for hornets to navigate the nest.

The hexagonal shape of Asian hornet comb cells allows for a regular, gap-free division of a plane surface. Although other shapes, such as squares and triangles, can achieve this, the hexagon encloses the same area with the least circumference. As a result, hexagonal cells require less adhesion material than triangular or square cells with the same volume, making them more economical [3]. Additionally, the hexagonal shape is better suited for the growth of plump Asian hornet larvae inside the cells. When the flat surface of the comb is curved in one plane, the shapes of the hexagons, squares, and triangles also become curved. This effect can be observed by rolling a piece of squared paper into a tube. However, if the surface is curved in two planes to form a dome, no single regular identical unit is suitable, and each unit must be individually adjusted. The Asian hornet is distinguished by its distinctive comb cells, which are large and elongated. Section 3.3.2 of this paper provides a comprehensive analysis of the measurements taken on Asian hornet comb cells, including detailed information on their size, shape, and orientation.

The space between the combs in an AHN serves several pivotal functions essential for colony survival and proliferation. These functions include insulation, ventilation, movement, and expansion:

- Insulation plays a crucial role in the space between the combs, as it helps maintain a stable temperature within the nest [39]. The insulating function of the space between the combs ensures the optimal conditions necessary for the colony’s continued growth and success. This is especially critical for protecting the developing hornet larvae from exposure to extreme temperatures that could prove detrimental to their survival [34].
- Ventilation is another fundamental function of the space between the combs, as it facilitates the regulation of temperature and humidity levels within the nest [36]. The space between the combs serves as an efficient means of achieving optimal ventilation, which is essential for maintaining colony homeostasis. Proper ventilation ensures the nest environment remains free from harmful substances that could compromise the colony’s health and well-being.
- Movement is also enabled by the space between the combs, providing a pathway for the adult hornets to attend to the needs of the developing larvae and eggs.
- Expansion refers to the space between the combs, which also allows for the expansion of the colony. As the colony grows, hornets can construct additional combs and add them to the existing space between the combs, ensuring that the colony can accommodate more members as needed [40].

#### The Pedicels and Adhesion Material

Figure 6 illustrates a 3D model of the internal structure of the AHN and reveals how the nest adheres to the tree branches. The model shows that the combs are interconnected and attached to the base (tree branch) through stalk-like structures called pedicels [41]. Pedicels, which are small tubes, columns, or pillars, support the comb cells in the AHN. These structures play a critical role in the nest’s structural framework by providing stability and support for the multiple comb layers within the AHN. The pedicels are composed of a tough material, primarily consisting of a secretion produced in the hornet’s mouth [38].

The aim of examining the pedicels’ structural properties would be to gain a deeper understanding of the stability and construction of the AHN, which could lead to the development of innovative biomimetic designs and engineering solutions that have practical applications. Depending on the level of the nest at which the pedicels are attached, their thickness and accumulation occur at varying rates in different areas of the nest. The accumulation of pedicels reinforces the nest structure and distributes its weight evenly across the tree branch, thus preventing it from breaking or detaching. The material used for adhesion, as well as the mode of attachment of the combs to the outer envelope and tree branches, also contribute to the structure’s overall stability by acting as pillars. As the comb layers increase in size and height, the number and thickness of pedicels also increase, ultimately resulting in the attachment of the nest to the tree branch at the top. Figure 6 illustrates the attachment of the nest to the tree branch, with Level 1 being the bottom comb and Level 5 being the top comb. The pedicels are integral elements of the nest structure, responsible for transferring the load from the bottom layer to the outer envelope and the tree branches, which provide a stable foundation. Therefore, strengthening the pedicels is essential to ensure the even distribution of the nest weight and to mitigate the risk of detachment or collapse. The findings of this study offer insights into the architectural and engineering principles that govern comb structure construction in social insects and have significant implications for the design of stable structures in other fields. A critical mechanical property of the pedicels is their capacity to withstand tensile strain, referring to the ability of the nest to resist deformation under tensile stress, which pulls on the material. The pedicels’ structure plays a crucial role in supporting tensile strain, helping to maintain the nest’s shape and stability in the face of external stressors. By comprehending the properties of pedicels and their contribution to Asian hornet nest stability, engineers can devise innovative solutions for constructing stable structures in various contexts.

Social wasps employ various thermoregulatory mechanisms, such as fanning their wings to enhance them and “curling” behaviour, in which the queen curls her body around the pedicels of the nest [42,43]. The pedicels also enable effective temperature regulation within the nest [37]. The pedicels reinforce these mechanisms by providing a narrow attachment point for the nest cells, improving air circulation throughout the structure. This augmented airflow reduces temperatures within the nest, ensuring a hospitable living environment for the hornets.

In the Vespa velutina nigrithorax’s nest, the pedicels are long enough to allow the clear passage of hornets from one comb to another. The pedicel, for instance, functions as a barrier against walking insects and reduces the contact surface area between the nest and the substrate to a minimum. This reduction allows hornets to easily monitor the small potential invasion zone and quickly respond to threats [44,45].

The investigation of the adhesion material used by hornets to attach their nest to the tree branches is not only interesting from a biological point of view but also from the point of view of the materials science and engineering fields. The hornet’s ability to produce a highly adhesive and durable material with local and limited resources is a great inspiration for developing sustainable and eco-friendly building materials.

### 3.3. Other Important Features of the Asian Hornet Nest

#### 3.3.1. Centre of Gravity in the Asian Hornet Nest

**Figure 7:**
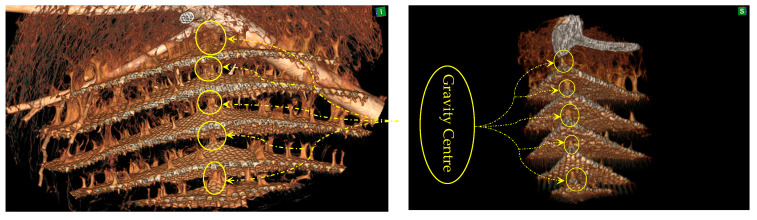
Column support and centre of gravity in the AHN comb, the patient orientation in space: (S)—superior and (I)—inferior.

In engineering, the centre of gravity is a fundamental concept in determining the stability and equilibrium of structures. The location of the centre of gravity in relation to the base of support is crucial for both static and dynamic stability, as it determines the ability of an object to maintain its balance and prevent it from falling over [46]. Hornet combs, as complex structures, follow the same principle and rely on the placement of the centre of gravity to ensure their balance. The centre of gravity of a hornet comb denotes the point at which the weight of the comb is evenly distributed in all directions, and it is a critical determinant of the comb’s overall stability, as shown in Figure 7. The strategic placement of columns within the comb structure around the centre of gravity significantly contributes to enhancing the comb’s stability and balance. This design element ensures that any external forces acting on the comb are efficiently distributed, thereby minimising the likelihood of collapse. Furthermore, the columns reinforce the structure and enhance its resistance to extra loads.

The hornets’ remarkable ability to comprehend physics and engineering principles is awe-inspiring. Placing columns at the centre of gravity in the comb structure is a testament to their intelligence and ingenuity. This design strategy allows hornet combs to remain securely attached to their supports.

#### 3.3.2. The Outer Envelope and the Combs’ Design and Measurements after the CT Scan

This study examined five combs within an AHN, measuring their respective maximum ($L_{C}{}_{max}$) and minimum ($L_{C}{}_{min}$) lengths using a CT scan (Table 1). The area of each comb (*A_cb_*) was estimated using the standard mathematical formula for the area of an ellipsoid, assuming that their shapes were elliptical and similar, as shown in Equation (2).

(2) $$A_{cb}=\pi \cdot \left[\left(L_{C}{}_{max}/2\right)\cdot \left(L_{C}{}_{min}/2\right)\right]$$

**Figure 8:**
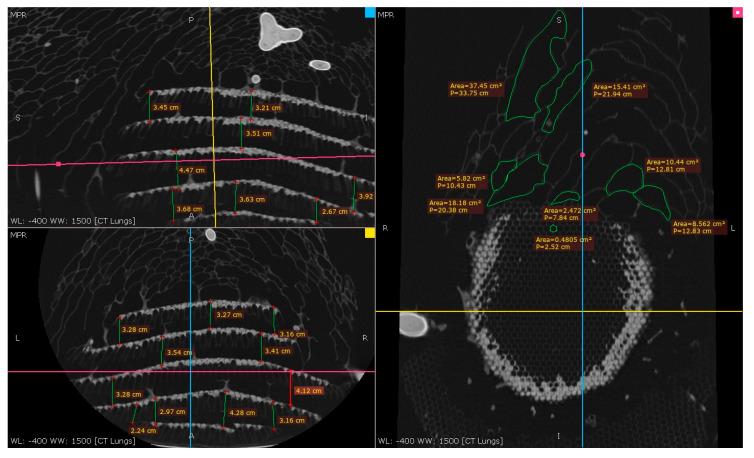

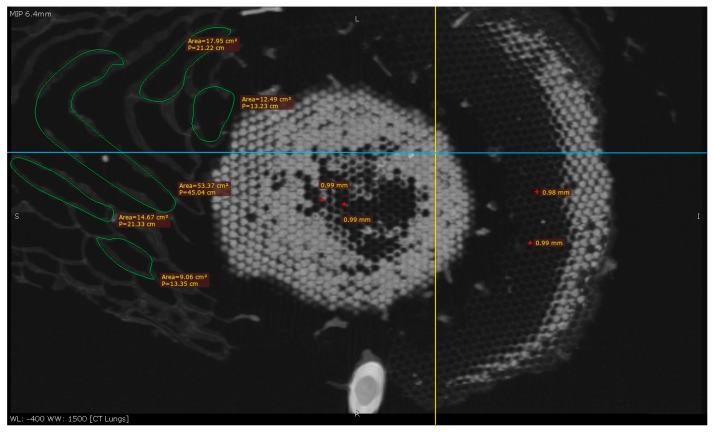
Asian hornet nest measurement using CT scan.

**Figure 9:**
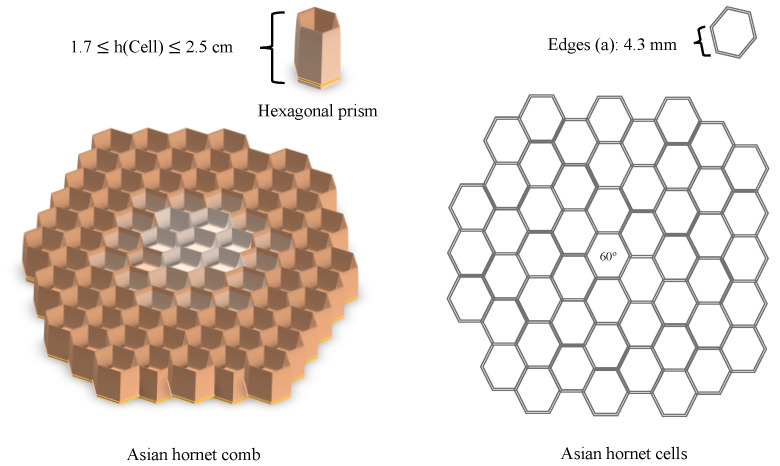
Cell sizes of the Asian hornet nest combs.

This section presents the measurements of the comb’s cells, including the thickness of the walls (0.99 mm), the design and size of the hexagonal prism, the area of cells, and the volume of the hexagonal prism. The measurements of the nest combs are as follows: the base edge of the hexagonal prism shape (*a*) measured 4.3 mm, and the height (*h*) ranged from 17 mm to 25 mm, as illustrated in Figure 8 and Figure 9.

- Area of the cell: $Ac=\frac{3\sqrt{3}}{2}(a^{2})$= 0.4804 cm^2^.
- Volume of the hexagonal prism: $\left(V\right)=\frac{3\sqrt{3}}{2}(a^{2})\;\left(h\right),$ = 0.82 cm^2^ ≤ (*V*) ≤ 1.201 cm^2^, depending on the hexagonal prism height (*h*).

As illustrated in Figure 8, the CT scan was also employed to determine the area of the cavities that formed the outer envelope, where larger cavities were situated farther from the combs and smaller ones were in closer proximity.

To calculate the number of cells in each comb, a simple computation is required by dividing the area of the comb by the area of each individual cell, assuming that each cell is of equal size and shape. Although this estimate may not be entirely precise, any discrepancies between the estimated and actual cell numbers are likely negligible.

**Figure 10:**
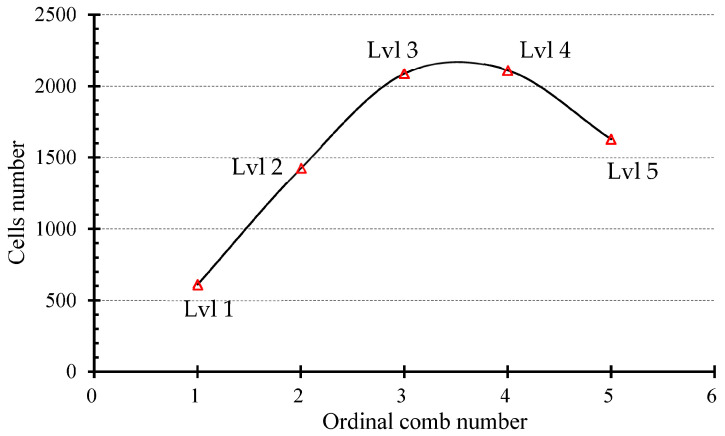
Number of cells by ordinal comb number.

Figure 10 presents a graphical representation of the number of cells in each comb of the Asian hornet nest, ordered by comb level. The graph illustrates a gradual increase in the number of cells from the Level 1 comb to the Level 3 comb, followed by a plateau in the Level 4 comb, which has a similar area and cell count to Level 3 (2088 and 2111 cells, respectively). The number of cells then decreases again in the Level 5 comb to a value of 1628 cells.

The observed variation in cell count across the different comb levels can be interpreted as a strategic building process by the hornets. The primary comb at Level 5 is constructed with a smaller area and fewer cells, after which the hornets build larger combs (Level 3 and Level 4) with more cells to expand the nest. The size and cell count in Levels 1 and 2 are then reduced to enhance nest stability and prevent detachment. This variation in cell count is a crucial design feature for the structural integrity of the nest. It is imperative that the nest remain stable during extreme weather events or when exposed to external forces. The hornets’ strategic building process is a prime example of how engineering principles are inherent in nature and can be applied to design durable and robust structures.

**Table 1:** Geometrical properties of the Asian hornet nest.

	Comb Levels	1st Level	2nd Level	3rd Level	4th Level	5th Level
Parameters						
$L_{C}{}_{max}$ (cm)		19.5	29.7	37.6	36.9	33.3
$L_{C}{}_{min}$ (cm)		17.3	28.8	34.3	34.4	30.6
$\mathrm{Area}\;\mathrm{calculated}\;(A_{cb}$) (cm^2^)		265	672	1013	997	800
Area from CT scan		293	686	1003	1014	782
Number of cells (from CT scan)		610	1428	2088	2111	1628
Number of cells (calculated)		552	1398	2108	2075	1666
Volume from CT (cm^3^)		615	1440	2106	2129	1643
Volume calculated * (cm^3^)		556	1411	2127	2094	1681
		Figure 11f	Figure 11c	Figure 11d	Figure 11c	Figure 11b

* The mean height was adopted to calculate the volume of each comb (21 mm).

**Figure 12:**
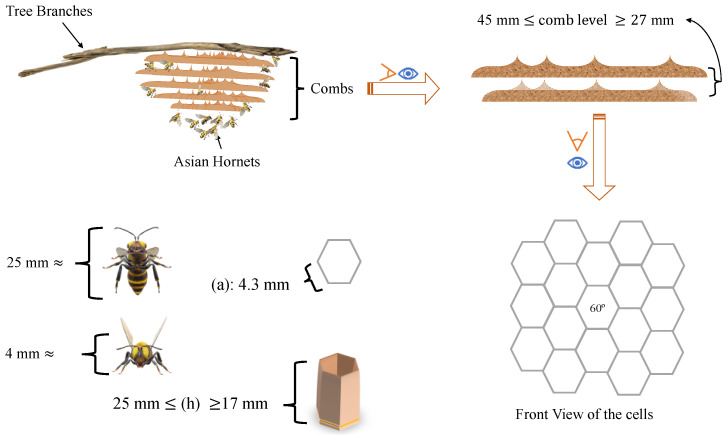
A schematic diagram of the Asian hornet nest after examination by CT scan, with more information about the Asian hornet size.

Through the use of CT scan technology, we can create a schematic diagram of the Asian hornet nest after a comprehensive examination as it is shown in Figure 12.

### 3.4. X-ray Analysis

The resultant X-ray image, referred to as X-ray radiograph or simply radiograph, is created by passing X-rays through the AHN and capturing the resulting transmission of radiation on a digital detector. This process generates a monochromatic image that displays the internal structure of the Asian wasp nest, with denser materials, such as the tree branch and the lower edge of the combs, appearing in bright white. In contrast, less dense materials, such as soft tissue (cells of combs and the outer envelope), appear darker.

**Figure 13:**
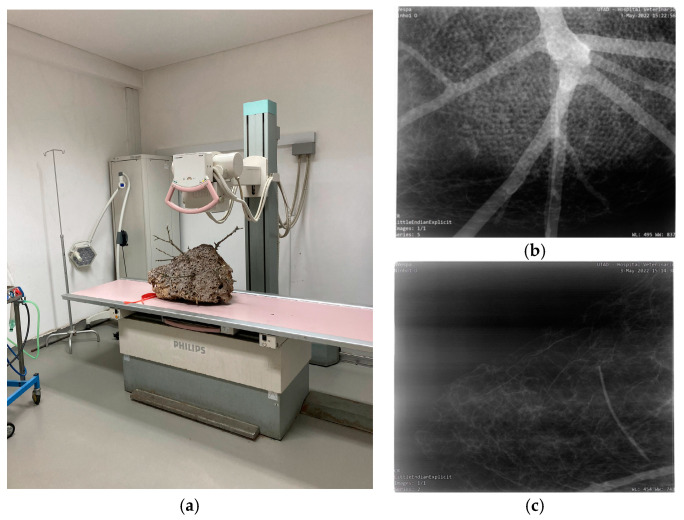
Radiographs of the AHN: (**a**) position of the nest during the X-ray scanning; (**b**) X-ray image of the tree branch and cells; (**c**) X-ray image of the outer envelope [47].

**Figure 14:**
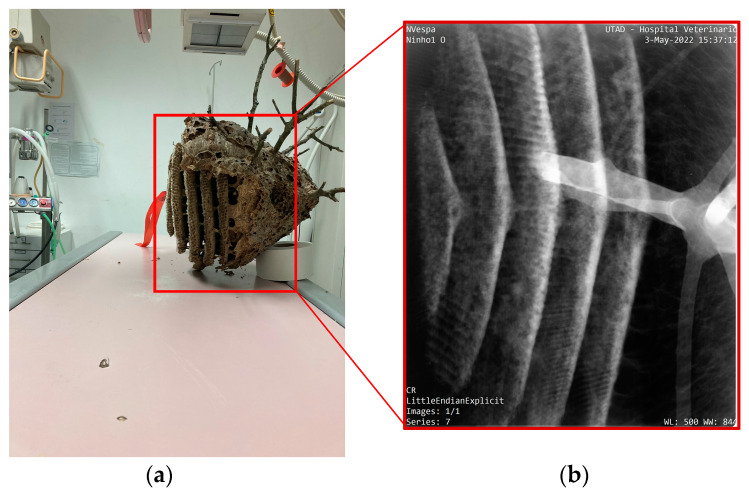
Radiographs of the AWN: (**a**) position of the nest during the X-ray scanning; (**b**) X-ray image of the comb layers.

Multiple X-ray images taken from varying angles highlight the intricate connections between the tree branch and the AHN (Figure 13b and Figure 14b). Notably, owing to its higher density, the tree branch exhibits a more distinct bright white colouration, as evidenced by the X-ray analysis results. These findings convincingly illustrate the connection between the nest and the tree branch. Moreover, the X-ray images allow for the identification of the various comb layers of the nest, as shown in Figure 14a. Intriguingly, the Asian hornet’s nest consists of five symmetrical combs, a fact that can be ascertained using X-ray radiographs.

### 3.5. Comparing Results of CAT Scans and X-rays: An Analysis

CT scans and X-ray analyses are two radiological imaging techniques used to examine the internal structure of Asian hornet nests non-destructively. Both techniques utilise X-rays to penetrate the object and create images of its internal structure.

CT scans use advanced techniques such as 3D volume rendering, and the “Angio” parameter refines the CT scan images for a comprehensive understanding of the nest’s internal structure. X-rays produce a two-dimensional image of the nest’s internal structure from a specific angle, allowing for the identification of different comb layers and intricate connections between the nest and the tree branch. While both techniques have advantages and disadvantages, CT scans provide a more detailed and comprehensive view of the internal structure of the hornet nest, including a 3D reconstruction. At the same time, X-ray analysis is less time-consuming and less expensive, but it only provides a 2D image of the internal structure. However, X-rays have limited ability to distinguish between materials with similar densities, making it challenging to differentiate between the layers that make up the outer envelope and the pedicels of the Asian hornet nest. In contrast, CT scans differentiate between materials with similar densities, ensuring a more accurate assessment of the different layers that make up the Asian hornet nest, including the outer envelope.

CT scans reveal the structural components of the nest, such as the arrangement and shape of the combs, which resemble mushrooms with an umbonate pileus. The circular, disc-shaped, and gravity-centred combs distribute the weight of the nest evenly, reducing stress on the overall structure. Additionally, CT scans offer several advantages over X-rays when it comes to examining the outer envelope of AHNs. CT scans utilise a series of X-ray images taken from multiple angles and combine them to create a detailed 3D image of the object being scanned. This allows for a more accurate assessment of the different layers that make up the AHN, including the outer envelope. CT scans also offer a more detailed image of the nest, facilitating a more comprehensive understanding of its structure and function. Another advantage of CT scans is their ability to generate images of the AHN in different planes, such as coronal or sagittal. This offers additional information about the shape and orientation of the comb inside the outer envelope and any abnormalities or irregularities that may be present.

## 4. Scanning Electron Microscopy (SEM) with Energy Dispersive Spectroscopy (EDS) Analysis of the Asian Hornet Nest

### 4.1. SEM Analysis

**Figure 15:**
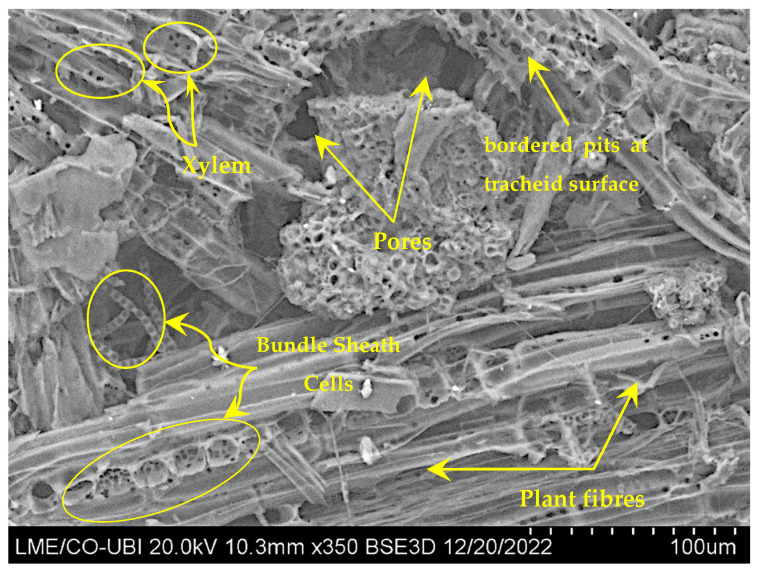
SEM images of the outer envelope of the Asian hornet nest’s paper-like material.

Asian hornet combs are enveloped with a paper-like material made of plant fibres and oral secretions, which is primarily derived from sieve tubes or vessels in plant tissues. These vessels provide a natural source of material for the nest envelope, and oral secretions help to bind the fibres together [27]. This envelope has grooves on its surface that are used to manipulate and apply the plant fibres, as well as numerous pores that regulate humidity and temperature in the nest. In addition, this provides the hornet with a comfortable environment for its larvae, which are dependent upon a certain temperature and humidity in order to develop and grow properly [36]. The pores (mesopores) also allow for air circulation, which helps to keep the nest clean and protected from potential pathogens. The SEM image in Figure 15 shows the presence of palisade parenchyma mesophyll cells and bundle sheath strands, which are components of the leaf structure. The Asian hornet nest also uses xylem tissue, made up of the tracheid’s cells and vessels, to construct the nest. The mesophyll cells are arranged in a palisade layer, which provides the leaf with a large surface area to absorb light for photosynthesis. The bundle sheath strands comprise tightly packed cells that surround the veins and transport nutrients throughout the leaf. The xylem tissue provides structural support for the nest by forming a rigid outer layer. The xylem tissue is responsible for transporting water and minerals from the roots of the plant to its leaves [48,49]. Also, there is the presence of bundle sheath strands isolated from crabgrass leaves and bordered pits on the tracheid surface. Bundle sheath strands are specialised cells that surround the veins in the leaves of some plants [50,51]. Overall, the nest materials of Asian hornets exhibit complex microstructures that contribute to their functionality and ability to regulate the internal environment for survival.

### 4.2. EDS Analysis

The EDS analysis conducted on the paper-like material provides the chemical composition of the material that hornets used to build up the nest. The paper-like material analysed includes elements such as carbon (52.74 mass.%), oxygen (45.02 mass.%), aluminium (1.33 mass.%), potassium (0.12 mass.%), and calcium (0.52 mass.%), as shown in Table 2. The chemical composition offers a deeper understanding of the type of materials that the Asian hornets used during their nest construction process. Additionally, it can also be used to detect any contaminants that may have been introduced to the nest.

Cellulose, hemicellulose, and lignin are lignocellulosic materials made up of C and O elements. Cellulose is composed of long chains of sugar molecules that are held together by strong bonds between the carbon and oxygen atoms. Hemicellulose is composed of shorter chains of sugar molecules, while lignin is composed of complex polyphenolic molecules [52,53]. Cellulose is a polysaccharide, which means it is composed of many large carbohydrate molecules that are linked together. Hemicellulose is a more complex polysaccharide with shorter chain lengths, and lignin is a non-carbohydrate molecule made of aromatic compounds. These molecules give wood-based materials their strength and rigidity [54].

In a comparative study, a difference was noted in the chemical makeup of the Asian hornet nest paper when compared to N. Crespo et al.’s [55] research on the same hornet species in a comparable area. N. Crespo et al. found that the hornets employed 99% organic matter (C and O), whereas the hornets in this study utilised 97.76% organic matter (C and O). These outcomes imply that it is possible that the hornets in this study lived in different environmental conditions, which could explain the small discrepancy in the amount of organic matter present in their nests.

The construction of Asian hornet nests involves the use of paper-like material gathered from the surrounding environment, including wood and tree leaves. As such, it is possible for inorganic elements to be present in the nests due to the potential contamination of this material during the construction process (Al, Si, K, and Ca). These contaminants may come from the surrounding environment, where the materials were originally sourced, and have the potential to affect the chemical composition of the Asian hornet nest. Therefore, it is important to consider potential sources of contamination when analysing the chemical composition of the Asian hornet nest paper. It is also possible that the difference in organic matter content could be due to natural variations in the environment or differences in the availability of materials used to build the nest. Further studies may be necessary to fully understand the factors that contribute to variations in the chemical makeup of AHNs’ paper-like material.

## 5. Conclusions

In conclusion, this study of AHNs offers a deep understanding of the materials, design, and engineering principles used by hornets to create their intricate structures. The key findings of this research contribute to the understanding of the complex architecture of these nests, which serve as both shelter and home to the hornets. The construction process of the Asian hornet nest, completed in a notably short timeframe considering the builders’ size, serves as a source of inspiration for the construction and materials industries.

The AHN’s outer envelope has a vital, multifaceted role in protecting, regulating, and supporting the nest, making it crucial for colony survival and development.

The disc-shaped combs serve an essential role in distributing the weight of the nest more evenly, and the space between the combs fulfils various significant functions such as insulation, ventilation, movement, and expansion. This comprehension of the functional morphology of the nest shows the specific adaptations that enable these hornets to survive and thrive in their natural environment. The adhesion of the nest to the tree branches is critical to preventing collapse, and the pedicels in the nest serve as critical structural components, providing stability and support for the different levels of combs. These findings offer important insights into the biomechanics of the nest and the complex interplay between its different components.

The strategic placement of columns around the centre of gravity is essential for ensuring the stability of the combs, and the hornets’ adhesive material has the potential to inspire sustainable building materials. This finding has important implications for the field of biomimicry, which seeks to develop new and sustainable technologies based on natural designs and systems.

Both X-rays and CT scans facilitate the non-destructive testing of materials and structures. While both techniques are valuable, CT scans offer several advantages over X-rays in terms of examining the outer envelope and pedicels of AHNs, making them a superior tool for evaluating the structure and function of nests.

SEM-EDS is a reliable method to acquire detailed information on the microstructure and the chemical composition of nests’ paper-like material. Plant fibres and oral secretions are used to make the paper-like cover of Asian hornet combs. As a natural source of material for the nest envelope, oral secretions help bind the fibres.

The exceptional architectural design, rapid construction abilities, adhesive materials, and unique material properties found in these nests have the potential to significantly transform the civil engineering field, leading to more efficient and sustainable solutions in the construction sector.

This research demonstrates hornets’ exceptional intelligence and ingenuity in creating their nests and the potential for biomimicry in developing sustainable and eco-friendly materials and structures. These findings carry substantial implications for understanding insect behaviour and evolution, as well as for the development of new civil engineering and architectural technologies inspired by the natural world.

## Figures and Tables

**Figure 11:**
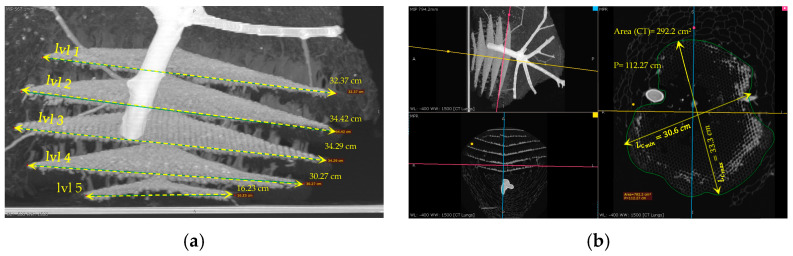

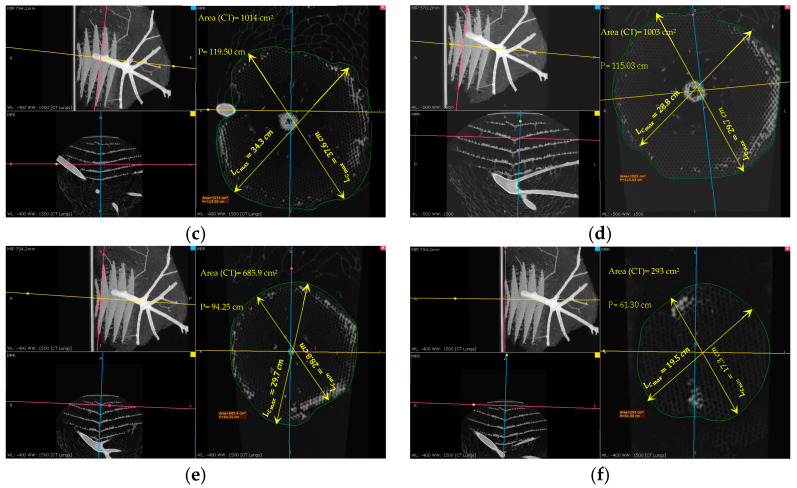
Three-dimensional multiplanar reconstructions (MNRs) of the Asian hornet nest with measurements (**a**–**f**).

**Table 2:** Chemical composition of the paper-like material in the Asian hornet nest comb.

Elements	Mass (%)
C	52.74
O	45.02
Al	1.33
Si	0.27
K	0.12
Ca	0.52

## Data Availability

Not applicable.
